# 5-{2-[(2-Hydroxy-5-methyl­phen­yl)(phenyl)methyl­eneamino]phenyl­imino­meth­yl}­pyrrole-2-carbaldehyde

**DOI:** 10.1107/S1600536808021922

**Published:** 2008-07-19

**Authors:** Ge Liu, Jianhui Wang, Liqin Guo, Wen-Juan Ruan

**Affiliations:** a Department of Chemistry, Nankai University, Tianjin 300071, People’s Republic of China; b Chifeng University, Chifeng 024000, People’s Republic of China

## Abstract

The title compound, C_26_H_21_N_3_O_2_, is an unsymmetrical tetra­dentate Schiff base ligand. The hydr­oxy group forms an intra­molecular O—H⋯N hydrogen bond with an adjacent N atom. An inter­molecular N—H⋯O hydrogen bond creates centrosymmetric dimers in the crystal packing.

## Related literature

For background, see: Ainscough *et al.* (1995 ▶); Aruffo *et al.* (1984 ▶). For further synthetic details, see: Atkins *et al.* (1985 ▶); Miller & Olsson (1981 ▶); Olsson & Pernemalm (1979 ▶); Zhu *et al.* (2004 ▶).
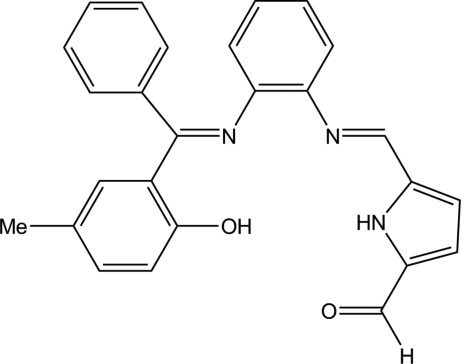


## Experimental

### 

#### Crystal data

C_26_H_21_N_3_O_2_
*M* *_r_* = 407.46
Triclinic, 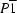

*a* = 8.8299 (18) Å
*b* = 9.4816 (19) Å
*c* = 13.130 (3) Å
α = 94.05 (3)°
β = 106.32 (3)°
γ = 94.88 (3)°
*V* = 1046.0 (4) Å^3^
*Z* = 2
Mo *K*α radiation
μ = 0.08 mm^−1^
*T* = 113 (2) K
0.22 × 0.16 × 0.12 mm

#### Data collection

Rigaku R-AXIS RAPID-S diffractometer
Absorption correction: multi-scan (*CrystalClear*; Rigaku/MSC, 2001 ▶) *T*_min_ = 0.98, *T*_max_ = 0.99
10760 measured reflections
3695 independent reflections
3065 reflections with *I* > 2σ(*I*)
*R*_int_ = 0.042

#### Refinement

*R*[*F* ^2^ > 2σ(*F* ^2^)] = 0.047
*wR*(*F* ^2^) = 0.129
*S* = 1.08
3695 reflections
286 parameters
H atoms treated by a mixture of independent and constrained refinement
Δρ_max_ = 0.18 e Å^−3^
Δρ_min_ = −0.25 e Å^−3^

### 

Data collection: *RAPID-AUTO* (Rigaku, 1998 ▶); cell refinement: *RAPID-AUTO*; data reduction: *CrystalStructure* (Rigaku/MSC, 2002 ▶); program(s) used to solve structure: *SHELXS97* (Sheldrick, 2008 ▶); program(s) used to refine structure: *SHELXL97* (Sheldrick, 2008 ▶); molecular graphics: *SHELXTL* (Sheldrick, 2008 ▶); software used to prepare material for publication: *SHELXTL*.

## Supplementary Material

Crystal structure: contains datablocks global, I. DOI: 10.1107/S1600536808021922/bg2193sup1.cif

Structure factors: contains datablocks I. DOI: 10.1107/S1600536808021922/bg2193Isup2.hkl

Additional supplementary materials:  crystallographic information; 3D view; checkCIF report

## Figures and Tables

**Table 1:** Hydrogen-bond geometry (Å, °)

*D*—H⋯*A*	*D*—H	H⋯*A*	*D*⋯*A*	*D*—H⋯*A*
N3—H3*A*⋯O2^i^	0.95 (2)	1.98 (2)	2.902 (2)	164.2 (18)
O1—H1⋯N1	0.82	1.81	2.536 (2)	147

Symmetry code: (i) 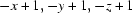.

**Fig. 1.**
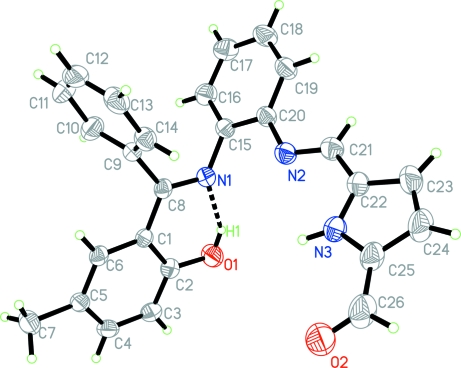
The structure of the title compound with 30% displacement probability.

**Fig. 2.**
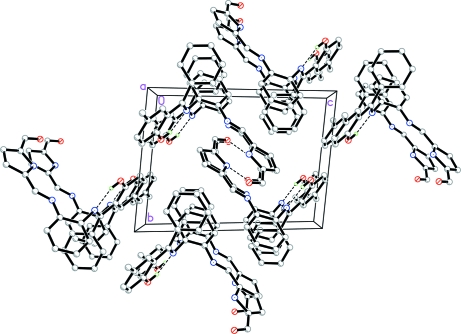
Crystal packing of the title compound, showing dimers piled along a. Hydrogen bonds shown as dashed lines.

## supplementary crystallographic information

### Comment

Unsymmetrical Schiff base ligands have been widely investigated due to their structural versatility; specially their metal complexes have been of interest to chemists (Aruffo *et al.*, 1984; Ainscough *et al.*,1995). In the course of the synthesis of one such a complex (Zhu *et al.*, 2004; Atkins *et al.*,1985), single crystals of the title compound C_26_H_21_N_3_O_2_ (I) were obtained, and its crystal and molecular structure is reported here (Fig.1). An intermolecular N—H···O hydrogen bond is formed between the *H*(pyrrole) atom of one molecule and *O*(aldehyde) of an adjacent molecule (Table 1), giving raise to centrosymmetric dimers in the crystal packing, piled as columnar arrays along a, as shown in Fig. 2

Moreover, the hydroxy group is involved in an intramolecular O—H···N hydrogen bond (Table 1, Fig.1), though which atoms O1, H1, N1, C8, C1 and C2 form a six-membered ring.

### Experimental

To a solution of 2-[(2-Aminophenyl)(phenyl)methyl]-4-methylphenol (0.2 mmol)(Atkins *et al.*,1985) in toluene (20 ml) was added pyrrole-2,5-dicarboxaldehyde (0.2 mmol)(Miller & Olsson, 1981; Olsso & Pernemalm, 1979) the mixture was stired and refluxed for two hours, then cooled. Rotary evaporation of solvent yielded the crude product; after chromatographic fractionating, it was recrystallized from the mixture of dichloromethane and hexane. Orange columnar crystals were obtained by evaporating the solvent at room temperature for about a week. yield: 53%, mp = 175°. Anal. for (C_26_H_21_N_3_O_2_), Calc. C, 76.64; H, 5.19; N, 10.31; Found: C, 76.12; H, 5.62; N, 10.19.

### Refinement

The H atoms (except H3A attached to N3) were positioned geometrically and allowed to ride on their parent atoms, with C—H=0.93Å and *U*_iso_(H)=1.2*U*_eq_(C) for the aromatic and pyrrole ring H atoms, C—H=0.96Å, and *U*_iso_(H)=1.5*U*_eq_(C) for the methyl H atoms, O-H: 0.82Å, *U*_iso_(H)=1.5*U*_eq_(O). H3A was found in the difference Fourier and refined freely with isotropic displacement parameters.

### Crystal data

**Table:**

C_26_H_21_N_3_O_2_	*Z* = 2
*M**_r_* = 407.46	*F*_000_ = 428
Triclinic, *P*1	*D*_x_ = 1.294 Mg m^−^^3^
Hall symbol: -P 1	Mo *K*α radiation λ = 0.71073 Å
*a* = 8.8299 (18) Å	Cell parameters from 3625 reflections
*b* = 9.4816 (19) Å	θ = 2.2–27.9º
*c* = 13.130 (3) Å	µ = 0.08 mm^−^^1^
α = 94.05 (3)º	*T* = 113 (2) K
β = 106.32 (3)º	Block, orange
γ = 94.88 (3)º	0.22 × 0.16 × 0.12 mm
*V* = 1046.0 (4) Å^3^	

### Data collection

**Table:**

Rigaku R-AXIS RAPID-S diffractometer	3695 independent reflections
Radiation source: fine-focus sealed tube	3065 reflections with *I* > 2σ(*I*)
Monochromator: graphite	*R*_int_ = 0.042
*T* = 113(2) K	θ_max_ = 25.0º
ω scans	θ_min_ = 1.6º
Absorption correction: multi-scan(Crystalclear; Rigaku/MSC, 2001)	*h* = −10→10
*T*_min_ = 0.98, *T*_max_ = 0.99	*k* = −11→11
10760 measured reflections	*l* = −15→15

### Refinement

**Table:**

Refinement on *F*^2^	Secondary atom site location: difference Fourier map
Least-squares matrix: full	Hydrogen site location: inferred from neighbouring sites
*R*[*F*^2^ > 2σ(*F*^2^)] = 0.047	H atoms treated by a mixture of independent and constrained refinement
*wR*(*F*^2^) = 0.129	*w* = 1/[σ^2^(*F*_o_^2^) + (0.0642*P*)^2^ + 0.0663*P*] where *P* = (*F*_o_^2^ + 2*F*_c_^2^)/3
*S* = 1.08	(Δ/σ)_max_ < 0.001
3695 reflections	Δρ_max_ = 0.18 e Å^−^^3^
286 parameters	Δρ_min_ = −0.25 e Å^−^^3^
Primary atom site location: structure-invariant direct methods	Extinction correction: none

### Special details

**Table:**

Geometry. All e.s.d.'s (except the e.s.d. in the dihedral angle between two l.s. planes) are estimated using the full covariance matrix. The cell e.s.d.'s are taken into account individually in the estimation of e.s.d.'s in distances, angles and torsion angles; correlations between e.s.d.'s in cell parameters are only used when they are defined by crystal symmetry. An approximate (isotropic) treatment of cell e.s.d.'s is used for estimating e.s.d.'s involving l.s. planes.
Refinement. Refinement of *F*^2^ against ALL reflections. The weighted *R*-factor *wR* and goodness of fit *S* are based on *F*^2^, conventional *R*-factors *R* are based on *F*, with *F* set to zero for negative *F*^2^. The threshold expression of *F*^2^ > σ(*F*^2^) is used only for calculating *R*-factors(gt) *etc*. and is not relevant to the choice of reflections for refinement. *R*-factors based on *F*^2^ are statistically about twice as large as those based on *F*, and *R*- factors based on ALL data will be even larger.

### Fractional atomic coordinates and isotropic or equivalent isotropic displacement parameters (Å^2^)

**Table:**

	*x*	*y*	*z*	*U*_iso_*/*U*_eq_	
O2	0.45524 (17)	0.34953 (15)	0.43121 (12)	0.0511 (4)	
O1	0.99294 (14)	0.64418 (13)	0.90770 (11)	0.0365 (3)	
H1	1.0207	0.6932	0.8657	0.055*	
N1	0.98930 (16)	0.84591 (15)	0.79037 (11)	0.0272 (4)	
N2	0.96097 (17)	0.77359 (15)	0.57380 (11)	0.0299 (4)	
N3	0.74047 (18)	0.54096 (16)	0.44365 (12)	0.0317 (4)	
C1	0.79083 (19)	0.80636 (17)	0.87844 (13)	0.0237 (4)	
C2	0.8592 (2)	0.68803 (18)	0.92457 (14)	0.0280 (4)	
C3	0.7899 (2)	0.61353 (18)	0.99065 (14)	0.0317 (4)	
H3	0.8377	0.5384	1.0237	0.038*	
C4	0.6508 (2)	0.65101 (18)	1.00712 (14)	0.0298 (4)	
H4	0.6055	0.5997	1.0510	0.036*	
C5	0.5756 (2)	0.76395 (18)	0.95990 (13)	0.0267 (4)	
C6	0.64834 (19)	0.84052 (18)	0.89718 (13)	0.0258 (4)	
H6	0.6013	0.9173	0.8663	0.031*	
C7	0.4228 (2)	0.8022 (2)	0.97928 (15)	0.0343 (4)	
H7A	0.4454	0.8515	1.0492	0.051*	
H7B	0.3529	0.7171	0.9741	0.051*	
H7C	0.3730	0.8625	0.9269	0.051*	
C8	0.86574 (19)	0.88993 (17)	0.81202 (13)	0.0243 (4)	
C9	0.79928 (19)	1.02221 (18)	0.77147 (13)	0.0251 (4)	
C10	0.8237 (2)	1.14705 (19)	0.83785 (15)	0.0357 (5)	
H10	0.8767	1.1484	0.9100	0.043*	
C11	0.7691 (2)	1.2706 (2)	0.79686 (17)	0.0438 (5)	
H11	0.7873	1.3548	0.8415	0.053*	
C12	0.6882 (2)	1.2690 (2)	0.69028 (18)	0.0430 (5)	
H12	0.6520	1.3518	0.6631	0.052*	
C13	0.6611 (2)	1.1440 (2)	0.62386 (16)	0.0373 (5)	
H13	0.6050	1.1424	0.5522	0.045*	
C14	0.71752 (19)	1.02080 (19)	0.66395 (14)	0.0296 (4)	
H14	0.7007	0.9372	0.6189	0.036*	
C15	1.07804 (19)	0.92923 (18)	0.73647 (13)	0.0260 (4)	
C16	1.1816 (2)	1.04669 (19)	0.79313 (15)	0.0324 (4)	
H16	1.1853	1.0730	0.8634	0.039*	
C17	1.2783 (2)	1.12397 (19)	0.74603 (15)	0.0345 (5)	
H17	1.3471	1.2016	0.7846	0.041*	
C18	1.2727 (2)	1.08574 (19)	0.64174 (15)	0.0345 (5)	
H18	1.3376	1.1376	0.6097	0.041*	
C19	1.1705 (2)	0.97020 (19)	0.58480 (15)	0.0324 (4)	
H19	1.1664	0.9464	0.5141	0.039*	
C20	1.07336 (19)	0.88838 (18)	0.63105 (14)	0.0270 (4)	
C21	0.9842 (2)	0.71074 (19)	0.49084 (14)	0.0328 (4)	
H21	1.0767	0.7388	0.4736	0.039*	
C22	0.8739 (2)	0.59846 (18)	0.42278 (14)	0.0313 (4)	
C23	0.8828 (2)	0.5293 (2)	0.32786 (15)	0.0392 (5)	
H23	0.9618	0.5477	0.2949	0.047*	
C24	0.7517 (2)	0.4276 (2)	0.29150 (15)	0.0407 (5)	
H24	0.7272	0.3655	0.2297	0.049*	
C25	0.6644 (2)	0.4354 (2)	0.36358 (14)	0.0349 (5)	
C26	0.5251 (2)	0.3451 (2)	0.36340 (16)	0.0431 (5)	
H26	0.4841	0.2765	0.3057	0.052*	
H3A	0.692 (2)	0.574 (2)	0.4957 (16)	0.049 (6)*	

### Atomic displacement parameters (Å^2^)

**Table:**

	*U*^11^	*U*^22^	*U*^33^	*U*^12^	*U*^13^	*U*^23^
O2	0.0440 (9)	0.0476 (9)	0.0613 (10)	−0.0052 (7)	0.0220 (8)	−0.0130 (7)
O1	0.0350 (8)	0.0358 (8)	0.0477 (9)	0.0133 (6)	0.0212 (6)	0.0145 (6)
N1	0.0279 (8)	0.0282 (8)	0.0270 (8)	0.0030 (6)	0.0109 (6)	0.0015 (6)
N2	0.0300 (8)	0.0288 (8)	0.0301 (9)	0.0000 (6)	0.0088 (7)	0.0015 (6)
N3	0.0325 (9)	0.0312 (9)	0.0298 (9)	0.0014 (7)	0.0081 (7)	−0.0021 (7)
C1	0.0251 (9)	0.0229 (9)	0.0213 (9)	0.0005 (7)	0.0054 (7)	−0.0016 (7)
C2	0.0259 (9)	0.0267 (10)	0.0310 (10)	0.0026 (7)	0.0089 (8)	−0.0016 (7)
C3	0.0347 (10)	0.0251 (10)	0.0361 (11)	0.0044 (8)	0.0102 (8)	0.0062 (8)
C4	0.0337 (10)	0.0254 (10)	0.0300 (10)	−0.0038 (7)	0.0111 (8)	0.0015 (7)
C5	0.0259 (9)	0.0262 (9)	0.0260 (10)	−0.0016 (7)	0.0072 (7)	−0.0040 (7)
C6	0.0263 (9)	0.0248 (9)	0.0241 (9)	0.0029 (7)	0.0046 (7)	−0.0013 (7)
C7	0.0324 (10)	0.0353 (11)	0.0369 (11)	0.0031 (8)	0.0132 (8)	0.0033 (8)
C8	0.0257 (9)	0.0243 (9)	0.0208 (9)	−0.0011 (7)	0.0058 (7)	−0.0034 (7)
C9	0.0230 (9)	0.0265 (9)	0.0287 (10)	0.0012 (7)	0.0124 (7)	0.0032 (7)
C10	0.0393 (11)	0.0315 (11)	0.0366 (11)	0.0023 (8)	0.0134 (9)	−0.0014 (8)
C11	0.0491 (13)	0.0261 (11)	0.0610 (15)	0.0041 (9)	0.0250 (11)	−0.0004 (9)
C12	0.0389 (12)	0.0387 (12)	0.0639 (15)	0.0158 (9)	0.0274 (11)	0.0234 (10)
C13	0.0315 (11)	0.0471 (12)	0.0398 (11)	0.0125 (9)	0.0156 (9)	0.0159 (9)
C14	0.0245 (9)	0.0345 (10)	0.0316 (10)	0.0042 (7)	0.0105 (8)	0.0039 (8)
C15	0.0230 (9)	0.0266 (9)	0.0300 (10)	0.0062 (7)	0.0087 (7)	0.0047 (7)
C16	0.0331 (10)	0.0321 (10)	0.0304 (10)	0.0035 (8)	0.0075 (8)	0.0001 (8)
C17	0.0278 (10)	0.0330 (10)	0.0390 (12)	−0.0022 (8)	0.0059 (8)	0.0010 (8)
C18	0.0267 (10)	0.0361 (11)	0.0430 (12)	0.0007 (8)	0.0139 (9)	0.0068 (9)
C19	0.0312 (10)	0.0358 (11)	0.0323 (10)	0.0033 (8)	0.0136 (8)	0.0003 (8)
C20	0.0234 (9)	0.0274 (10)	0.0313 (10)	0.0034 (7)	0.0099 (8)	0.0019 (7)
C21	0.0310 (10)	0.0322 (11)	0.0372 (11)	0.0014 (8)	0.0135 (8)	0.0030 (8)
C22	0.0349 (10)	0.0291 (10)	0.0311 (11)	0.0042 (8)	0.0112 (8)	0.0024 (8)
C23	0.0422 (11)	0.0421 (12)	0.0354 (11)	0.0042 (9)	0.0160 (9)	−0.0019 (9)
C24	0.0433 (12)	0.0437 (12)	0.0316 (11)	0.0035 (9)	0.0082 (9)	−0.0073 (9)
C25	0.0321 (10)	0.0356 (11)	0.0308 (11)	0.0014 (8)	0.0015 (8)	−0.0043 (8)
C26	0.0355 (11)	0.0451 (12)	0.0410 (12)	−0.0003 (9)	0.0040 (10)	−0.0129 (9)

### Geometric parameters (Å, °)

**Table:**

O2—C26	1.218 (2)	C10—H10	0.9300
O1—C2	1.354 (2)	C11—C12	1.379 (3)
O1—H1	0.8200	C11—H11	0.9300
N1—C8	1.296 (2)	C12—C13	1.382 (3)
N1—C15	1.423 (2)	C12—H12	0.9300
N2—C21	1.280 (2)	C13—C14	1.389 (2)
N2—C20	1.420 (2)	C13—H13	0.9300
N3—C22	1.362 (2)	C14—H14	0.9300
N3—C25	1.378 (2)	C15—C20	1.399 (2)
N3—H3A	0.95 (2)	C15—C16	1.399 (2)
C1—C6	1.407 (2)	C16—C17	1.380 (3)
C1—C2	1.413 (2)	C16—H16	0.9300
C1—C8	1.469 (2)	C17—C18	1.378 (2)
C2—C3	1.392 (2)	C17—H17	0.9300
C3—C4	1.376 (2)	C18—C19	1.382 (3)
C3—H3	0.9300	C18—H18	0.9300
C4—C5	1.399 (2)	C19—C20	1.398 (3)
C4—H4	0.9300	C19—H19	0.9300
C5—C6	1.386 (2)	C21—C22	1.442 (3)
C5—C7	1.510 (2)	C21—H21	0.9300
C6—H6	0.9300	C22—C23	1.392 (2)
C7—H7A	0.9600	C23—C24	1.390 (3)
C7—H7B	0.9600	C23—H23	0.9300
C7—H7C	0.9600	C24—C25	1.381 (3)
C8—C9	1.498 (2)	C24—H24	0.9300
C9—C10	1.383 (2)	C25—C26	1.436 (3)
C9—C14	1.392 (2)	C26—H26	0.9300
C10—C11	1.391 (3)		
			
C2—O1—H1	109.5	C13—C12—H12	120.0
C8—N1—C15	121.20 (15)	C12—C13—C14	120.07 (19)
C21—N2—C20	118.13 (15)	C12—C13—H13	120.0
C22—N3—C25	108.68 (15)	C14—C13—H13	120.0
C22—N3—H3A	128.6 (12)	C13—C14—C9	120.12 (17)
C25—N3—H3A	121.9 (12)	C13—C14—H14	119.9
C6—C1—C2	117.98 (15)	C9—C14—H14	119.9
C6—C1—C8	121.21 (16)	C20—C15—C16	119.75 (16)
C2—C1—C8	120.80 (15)	C20—C15—N1	120.96 (15)
O1—C2—C3	117.93 (16)	C16—C15—N1	119.05 (15)
O1—C2—C1	122.09 (15)	C17—C16—C15	120.85 (17)
C3—C2—C1	119.98 (15)	C17—C16—H16	119.6
C4—C3—C2	120.01 (17)	C15—C16—H16	119.6
C4—C3—H3	120.0	C18—C17—C16	119.75 (17)
C2—C3—H3	120.0	C18—C17—H17	120.1
C3—C4—C5	122.01 (16)	C16—C17—H17	120.1
C3—C4—H4	119.0	C17—C18—C19	119.95 (18)
C5—C4—H4	119.0	C17—C18—H18	120.0
C6—C5—C4	117.52 (16)	C19—C18—H18	120.0
C6—C5—C7	121.70 (16)	C18—C19—C20	121.54 (17)
C4—C5—C7	120.76 (16)	C18—C19—H19	119.2
C5—C6—C1	122.41 (16)	C20—C19—H19	119.2
C5—C6—H6	118.8	C19—C20—C15	118.12 (16)
C1—C6—H6	118.8	C19—C20—N2	123.26 (16)
C5—C7—H7A	109.5	C15—C20—N2	118.41 (15)
C5—C7—H7B	109.5	N2—C21—C22	123.21 (17)
H7A—C7—H7B	109.5	N2—C21—H21	118.4
C5—C7—H7C	109.5	C22—C21—H21	118.4
H7A—C7—H7C	109.5	N3—C22—C23	108.20 (16)
H7B—C7—H7C	109.5	N3—C22—C21	123.94 (16)
N1—C8—C1	118.17 (15)	C23—C22—C21	127.86 (18)
N1—C8—C9	121.81 (15)	C24—C23—C22	107.35 (17)
C1—C8—C9	120.02 (14)	C24—C23—H23	126.3
C10—C9—C14	119.51 (17)	C22—C23—H23	126.3
C10—C9—C8	121.20 (16)	C25—C24—C23	107.77 (17)
C14—C9—C8	119.24 (15)	C25—C24—H24	126.1
C9—C10—C11	120.09 (18)	C23—C24—H24	126.1
C9—C10—H10	120.0	N3—C25—C24	108.00 (17)
C11—C10—H10	120.0	N3—C25—C26	123.92 (17)
C12—C11—C10	120.27 (18)	C24—C25—C26	127.93 (18)
C12—C11—H11	119.9	O2—C26—C25	126.53 (18)
C10—C11—H11	119.9	O2—C26—H26	116.7
C11—C12—C13	119.92 (19)	C25—C26—H26	116.7
C11—C12—H12	120.0		
			
C6—C1—C2—O1	177.55 (14)	C8—C9—C14—C13	−177.17 (15)
C8—C1—C2—O1	−1.8 (2)	C8—N1—C15—C20	−109.03 (19)
C6—C1—C2—C3	−3.2 (2)	C8—N1—C15—C16	76.5 (2)
C8—C1—C2—C3	177.49 (14)	C20—C15—C16—C17	0.7 (3)
O1—C2—C3—C4	−177.69 (15)	N1—C15—C16—C17	175.21 (16)
C1—C2—C3—C4	3.0 (3)	C15—C16—C17—C18	0.3 (3)
C2—C3—C4—C5	−0.6 (3)	C16—C17—C18—C19	−0.1 (3)
C3—C4—C5—C6	−1.6 (2)	C17—C18—C19—C20	−1.1 (3)
C3—C4—C5—C7	179.84 (15)	C18—C19—C20—C15	2.1 (3)
C4—C5—C6—C1	1.4 (2)	C18—C19—C20—N2	176.60 (16)
C7—C5—C6—C1	179.91 (15)	C16—C15—C20—C19	−1.8 (3)
C2—C1—C6—C5	1.0 (2)	N1—C15—C20—C19	−176.24 (15)
C8—C1—C6—C5	−179.68 (14)	C16—C15—C20—N2	−176.62 (15)
C15—N1—C8—C1	−173.10 (14)	N1—C15—C20—N2	9.0 (2)
C15—N1—C8—C9	6.8 (2)	C21—N2—C20—C19	24.6 (3)
C6—C1—C8—N1	−174.23 (14)	C21—N2—C20—C15	−160.85 (16)
C2—C1—C8—N1	5.1 (2)	C20—N2—C21—C22	−176.30 (16)
C6—C1—C8—C9	5.9 (2)	C25—N3—C22—C23	0.3 (2)
C2—C1—C8—C9	−174.80 (14)	C25—N3—C22—C21	−179.04 (17)
N1—C8—C9—C10	−104.20 (19)	N2—C21—C22—N3	−4.9 (3)
C1—C8—C9—C10	75.7 (2)	N2—C21—C22—C23	175.81 (18)
N1—C8—C9—C14	72.9 (2)	N3—C22—C23—C24	−0.2 (2)
C1—C8—C9—C14	−107.18 (17)	C21—C22—C23—C24	179.09 (19)
C14—C9—C10—C11	−1.1 (3)	C22—C23—C24—C25	0.1 (2)
C8—C9—C10—C11	176.07 (16)	C22—N3—C25—C24	−0.3 (2)
C9—C10—C11—C12	1.0 (3)	C22—N3—C25—C26	175.55 (19)
C10—C11—C12—C13	0.1 (3)	C23—C24—C25—N3	0.1 (2)
C11—C12—C13—C14	−1.1 (3)	C23—C24—C25—C26	−175.5 (2)
C12—C13—C14—C9	1.1 (3)	N3—C25—C26—O2	0.8 (3)
C10—C9—C14—C13	0.0 (2)	C24—C25—C26—O2	175.7 (2)

### Hydrogen-bond geometry (Å, °)

**Table:**

*D*—H···*A*	*D*—H	H···*A*	*D*···*A*	*D*—H···*A*
N3—H3A···O2^i^	0.95 (2)	1.98 (2)	2.902 (2)	164.2 (18)
O1—H1···N1	0.82	1.81	2.536 (2)	147

Symmetry codes: (i) −*x*+1, −*y*+1, −*z*+1.
